# A nomogram illustrating the probability of anastomotic leakage following cervical esophagogastrostomy

**DOI:** 10.1007/s00464-020-08107-0

**Published:** 2020-10-26

**Authors:** Joerg Lindenmann, Nicole Fink-Neuboeck, Christian Porubsky, Melanie Fediuk, Udo Anegg, Peter Kornprat, Maria Smolle, Alfred Maier, Josef Smolle, Freyja Maria Smolle-Juettner

**Affiliations:** 1 Division of Thoracic Surgery and Hyperbaric Surgery, Department of Surgery, Medical University of Graz, Auenbruggerplatz 29/3, 8036 Graz, Austria; 2 Department of General Surgery, Medical University of Graz, Graz, Austria; 3 Department of Orthopaedics and Trauma, Medical University of Graz, Graz, Austria; 4 Institute of Medical Informatics, Statistics and Documentation, Medical University of Graz, Graz, Austria

**Keywords:** Cervical esophagogastrostomy, Anastomotic leakage, Risk factors, Scoring, Prediction, Postoperative care

## Abstract

**Background:**

Early diagnosis of anastomotic dehiscence following cervical esophagogastrostomy may become difficult. Estimation of an individual probability could help to establish preventive and diagnostic measures. The predictive impact of epidemiological, surgery-related data and laboratory parameters on the development of anastomotic dehiscence was investigated in the immediate perioperative period.

**Methods:**

Retrospective study in 412 patients with cervical esophagogastrostomy following esophagectomy. Epidemiological data, risk factors, underlying disease, pre-treatment- and surgery-related data, C-reactive protein and albumin levels pre-and post-operatively were evaluated. We applied univariable and multivariable logistic regression analysis and developed a nomogram for individual risk assessment.

**Results:**

There were 345 male, 67 female patients, mean aged 61.5 years; 284 had orthotopic, 128 retrosternal gastric pull-up; 331 patients had carcinoma, 81 non-malignant disease. Mean duration of operation was 184 min; 235 patients had manual, 113 mechanical and 64 semi-mechanical suturing; 76 patients (18.5%) developed anastomotic dehiscence clinically evident at mean 11.4 days after surgery. In univariable testing young age, retrosternal conduit transposition, manual suturing, high body mass index, high ASA and high postoperative levels of C-reactive protein were predictors for anastomotic leakage. These six parameters which had yielded a *p* < 0.1 in the univariable analysis, were entered into a multivariable analysis and a nomogram allowing the determination of the patient’s individual risk was created.

**Conclusion:**

By using the nomogram as a supportive measure in the perioperative management, the patient’s individual probability of developing an anastomotic leak could be quantified which may help to take preventive measures improving the outcome.

Esophagectomy is the treatment of choice for curative therapy of non-metastasized esophageal cancer and for a variety of benign, mostly end-stage esophageal diseases, respectively [1]. Improvement in surgical techniques and perioperative management has markedly reduced complications following esophagectomy. Nevertheless, anastomotic leakage remains a common problem with an incidence varying between 6 and 41% [2, 3]. Impaired healing of the anastomosis is still a major cause of enhanced perioperative morbidity and mortality. The latter is lower in cervical esophagogastrostomy than in intrathoracic dehiscence [2, 4]. Regardless of the site of esophagogastrostomy, anastomotic leakage and its sequelae still affect both long-term quality of life and long-term survival, respectively. In this context, the early detection of anastomotic leakage still remains pivotal since delayed treatment is closely connected with prolonged hospital stay and enhanced morbidity [5–7].

Many risk factors of impaired anastomotic healing have been reported up to now. In this context, the route of reconstruction, the length of the gastric conduit, body mass index (BMI), comorbidities, active smoking history, surgical technique for the anastomosis and the time interval of resuming oral intake following esophagogastrostomy have been described [1, 5, 8–14]. In addition, postoperative laboratory parameters were used to predict the risk of anastomotic dehiscence [15–17].

However, early diagnosis of anastomotic dehiscence following cervical esophagogastrostomy remains still challenging. In most of the cases, neither by endoscopy nor by contrast swallow or by computed tomography (CT) scan detection of a small leakage is possible [18]. Preventive measures like ante-flexion of the neck in the postoperative period [19] or intentional delay of the postoperative oral intake have been suggested [10, 11]. Since there are numerous risk factors, the impact of predictive parameters and early intervention may serve as the most effective preventive actions for postoperative anastomotic leakage.

In this context, reliable estimation of the individual likelihood of developing an anastomotic leakage could help to establish both individually targeted preventive and diagnostic measures during the perioperative course. Therefore, we investigated the predictive impact of epidemiological, surgery-related data and laboratory parameters on the development of anastomotic dehiscence with the aim to develop a helpful nomogram calculating each patient´s individual probability.

## Materials and methods

We did a retrospective single-centre study in 412 consecutive patients who had cervical esophagogastrostomy following esophagectomy between 1/2004 and 12/2018. The present study was approved by the local Ethics committee (Nr. 30–367 ex 17/18). As this is a retrospective non-intervention study, the institutional review board waived the need for written informed consent from the patients.

The patient-specific data were collected prospectively in the database of our hospital and retrospectively extracted for statistical evaluation. Those medical records were reviewed for age, sex, BMI and ASA surgical risk classification (ASA-Physical status; American Society of Anaesthesiologists). Moreover, pulmonary, cardiovascular and renal comorbidity were recorded for each patient. The indication for esophagectomy, surgical approach, route of reconstruction, suturing technique, day of occurrence of leakage and pre- as well as postoperative plasma levels of C-reactive protein (CRP) and albumin were documented (Table 1).

**Table 1:** Characteristics of 412 patients undergoing esophagectomy with gastric pull-up and cervical esophagogastrostomy

Age (years)	61.7 ±11.9	21–88
Gender		
Male	67	16.3%
Female	345	83.7%
BMI	25.1 ± 4.3	15–41
Smoking	230	55.8%
Alcohol consumption	298	74.9%
COPD	99	25.4%
Impaired heart function	65	16.7%
Peripheral artery occlusive disease	43	11.1%
Renal insufficiency	36	9.3%
ASA		
1	19	5.2%
2	158	42.9%
3	160	43.5%
4	31	8.4%
Esophageal diagnosis		
Benign	81	19.7%
Malignant	331	80.3%
Induction therapy	107	26.0%
Duration of operation (minutes)	184 ± 82	93–545
Surgical approach		
Transhiatal	261	63.3%
Thoracoabdominal	151	36.7%
Route of transposition		
Orthotopic	284	68.9%
Retrosternal	128	31.1%
Suture technique		
Mechanical	113	27.4%
Semi-mechanical	64	15.6%
Manual	235	57.0%
Anastomotic dehiscence	76	18.4%
CRP		
Preoperative	19 ± 41	0.4–342
1st postoperative day	103 ± 62	7–440
3rd postoperative day	180 ± 78	2.7–436
Albumin		
Preoperative	4.0 ± 0.6	1.5–5.4
1st postoperative day	2.6 ± 0.6	1.2–5.7
3rd postoperative day	2.6 ± 0.4	1.3–5.4

*BMI* Body Mass Index, *COPD* chronic obstructive pulmonary disease, *ASA* American Society of Anesthesiologists, *CRP* C-reactive protein

Patients suffering from anastomotic leakage of the cervical esophagogastrostomy with corresponding clinical signs were consecutively included in this retrospective analysis. Those clinical signs were cervical wound inflammation or drainage, fever, elevated leucocytes and CRP levels. However, if anastomotic dehiscence was strongly suspected, the leak was visualized by flexible endoscopy. In ambiguous cases when the cervical wound presented unsuspicious, additional application of contrast medium or CT-scan was required to confirm the diagnosis.

Esophagectomy was routinely accomplished using one of the following two approaches. The transhiatal approach as described by Orringer was chosen in the case of benign underlying esophageal disease (i.e. end-stage achalasia, perforation, chronic stricture), in clinically node-negative T1 or T2 carcinoma and in the case of esophageal cancer of the distal third without evidence of para-esophageal lymph node involvement [20]. In the remaining cases, the thoracoabdominal approach according to McKeown was performed [21]. The minimally invasive approach (minimally invasive esophagectomy, MIE) applying both, thoracoscopy and laparoscopy, the latter with an additional, small utility incision, was done in those selected patients eligible for MIE. However, intrathoracic esophagogastrostomy according to Ivor-Lewis has not been used [4, 21–23].

Esophageal reconstruction after esophagectomy was done by gastric transposition with cervical esophagogastrostomy. The tubulated stomach was created using the stapler device, the width of the gastric conduit was determined with five centimetres in order to prevent postoperative broadening of the conduit. The staple line was oversewn using interrupted absorbable, monofilic 4/0 polydioxanone sutures (PDS®; Ethicon). The choice of transposition route was determined during surgery and depended on tumour extension and mediastinal lymph node involvement. When nodes were positive or there was tumour extension beyond the esophageal wall without contiguous organ invasion (where postoperative radiotherapy of the mediastinum might be considered), the retrosternal route was chosen to protect the conduit from external beam radiation. The posterior mediastinal (orthotopic) route was used for all other patients. A two-field lymphadenectomy (abdominal and mediastinal) was performed with transthoracic resection. In transhiatal resection, abdominal lymphadenectomy was performed with additional clearance of the lower posterior mediastinum as previously described [14, 24].

After gastric pull-up, cervical esophagogastrostomy was created using one of the three following techniques.

For manual anastomotic suturing, a double-layer posterior and a single-layer anterior wall technique using interrupted monofilic absorbable 4/0 polydioxanone sutures (PDS®; Ethicon) was applied as previously described [14]. This hand-sewn end-to-side anastomosis was used in the vast majority of cases until 2011 and even up to now especially reserved for crucial intraoperative situations namely in the presence of a short gastric conduit or in case of a brief residual cervical esophagus.

Semi-mechanical anastomosis was performed according to the technique described by Orringer [9]. Beside the lateral stay sutures at the staple line, the front lip of the anastomosis was oversewn with interrupted absorbable, monofilic sutures. This side-to-side anastomosis has been used routinely since 2012.

In the case of complete mechanical anastomosis, the circular stapler was used. Again, the entire staple line of this end-to-side anastomosis was oversewn with interrupted absorbable, monofilic sutures. Likewise this type of anastomosis has also been used since 2012.

After completion of the cervical esophagogastrostomy, a closed suction drain was placed adjacent to the anastomosis with the tip reaching towards the upper mediastinum. The cervical wound was closed in two layers using interrupted sutures. Before the abdomen was closed, a jejunal feeding tube was inserted enabling the start of early feeding of six hours after operation according to our established nutrition guidelines. All surgical interventions were performed by the same two teams of surgeons. As a routine, oral intake was gradually resumed, beginning with at least 7 days after surgery.

### Statistical analysis

Besides basic statistics, univariable logistic regression analysis was applied to each patient-specific parameter. Subsequently, all parameters which yielded a *p* value < 0.1 were included into a multivariable logistic regression analysis with a bootstrapping procedure, which in turn served as input for the generation of a nomogram [25] indicating the individual risk for developing anastomotic leakage and for ROC analysis. Statistical analyses were performed using Stata version 15 (Stata Corporation, College Station, Texas, USA).

## Results

The characteristics of those 412 patients undergoing esophagectomy with gastric pull-up and cervical esophagogastrostomy are displayed in detail in Table 1. A detailed subdivision of these patients focusing on gender, underlying esophageal disease, route of reconstruction, suture technique of the anastomosis and induction therapy is given in Table 2.

**Table 2:** Detailed subdivision of all 412 patients undergoing esophagectomy with gastric pull-up and cervical esophagogastrostomy

	Anastomotic leakage	
	No	Yes
Gender		
Male	277 (80%)	68 (20%)
Female	59 (88%)	8 (12%)
Esophageal carcinoma		
No	66 (81%)	15 (19%)
Yes	270 (82%)	61 (18%)
Orthotopic reconstruction		
No	91 (71%)	37 (29%)
Yes	245 (86%)	39 (14%)
Suture technique		
Manual	178 (76%)	57 (24%)
(Semi-)mechanical	158 (89%)	19 (11%)
Induction therapy		
No	247 (81%)	58 (19%)
Yes	89 (83%)	18 (17%)

Focused on gender, underlying esophageal disease, route of reconstruction, suture technique of the anastomosis and induction therapy in relation to the incidence of postoperative anastomotic dehiscence

76 patients (18.4%) developed anastomotic dehiscence. Anastomotic leakage became clinically evident at a mean of 11.4 days (3–30) after the operation. After manual suturing, anastomotic dehiscence could be detected in 24%, compared with 11% in semi-mechanical and mechanical anastomosis, respectively (Table 2). Univariable logistic regression analysis demonstrated a statistical significant relationship between the development of anastomotic leakage and both the used reconstruction route of the gastric conduit (*p* < 0.001) as well as the suture technique of the anastomosis itself (*p* < 0.001). In univariable analysis, neither the presence of esophageal malignancy nor the presence of induction therapy (preoperative chemotherapy and combined chemo-radiotherapy) had statistical significance on anastomotic dehiscence as given in Table 3.

**Table 3:** Univariable logistic regression analysis of all 412 patients undergoing esophagectomy with gastric pull-up and cervical esophagogastrostomy

	Odds ratio	Std. Err	z	P >|z|	95% Conf. Interval	
Age (years)	0.975	0.010	− 2.28	***0.023***	0.955	0.996
Male	1.810	0.724	1.48	0.138	0.826	3.967
BMI	1.051	0.031	1.65	***0.099***	0.990	1.115
Esophageal carcinoma	0.994	0.317	− 0.02	0.985	0.531	1.858
Duration of operation	1.002	0.001	1.37	0.171	0.999	1.005
Preoperative CRP	1.003	0.002	1.06	0.290	0.997	1.008
Preoperative Albumin	1.001	0.220	0.001	0.993	0.650	1.542
CRP 1st postoperative day	1.002	0.001	1.24	0.214	0.998	1.006
Albumin 1st postoperative day	0.906	0.216	− 0.41	0.680	0.567	1.447
CRP 3rd postoperative day	1.004	0.001	2.94	***0.003***	1.001	1.008
Albumin 3rd postoperative day	0.940	0.293	− 0.19	0.846	0.510	1.735
Manual suturing	2.662	0.763	3.42	***0.001***	1.518	4.669
Retrosternal conduit transposition	2.554	0.664	3.6	***0.001***	1.533	4.254
Induction therapy	0.865	0.267	− 0.47	0.641	0.472	1.587
Smoking	1.266	0.328	0.91	0.361	0.762	2.104
Alcohol consumption	1.102	0.134	0.8	0.423	0.867	1.401
COPD	1.148	0.250	0.63	0.527	0.748	1.761
Impaired heart function	0.911	0.328	− 0.26	0.797	0.449	1.848
Peripheral artery occlusive disease	1.695	0.640	1.4	0.163	0.808	3.555
Renal insufficiency	0.893	0.418	− 0.24	0.810	0.357	2.237
ASA	1.565	0.294	2.38	***0.017***	1.082	2.264

*BMI* Body Mass Index, *CRP* C-reactive protein, *COPD* chronic obstructive pulmonary disease, *ASA* American Society of Anaesthesiologists performance score

Bold-italic values are statistically significant for *P* values

Regarding the pre-and postoperative levels of CRP and Albumin, only the elevated levels of CRP on the third postoperative day yielded statistical significance (*p* < 0.003) using logistic regression analysis as displayed in Table 4.

**Table 4:** Logistic regression analysis of all 412 patients undergoing esophagectomy with gastric pull-up and cervical esophagogastrostomy focusing on the pre- and postoperative levels of CRP and Albumin in relation to the incidence of postoperative anastomotic dehiscence

	Anastomotic dehiscence		*p*
	No	Yes	
Preoperative CRP	18 ± 39	24 ± 48	0.296
CRP 1st postoperative day	102 ± 60	112 ± 69	0.218
CRP 3rd postoperative day	175 ± 76	206 ± 83	**0.003**
Preoperative Albumin	4.04 ± 0.64	4.05 ± 0.65	0.504
Albumin 1st postoperative day	2.61 ± 2.56	2.58 ± 0.58	0.677
Albumin 3rd postoperative day	2.56 ± 0.44	2.55 ± 0.36	0.857

*CRP* C-reactive protein

Bold values are statistically significant for *P* values

We entered each parameter which showed a *p* value < 0.1 in univariable logistic regression analysis into a multivariable logistic regression model of all 412 patients. These parameters were age, BMI, CRP on 3rd day, manual suturing, retrosternal conduit transposition and ASA score (Table 5).

**Table 5:** Multivariable analysis of all 412 patients undergoing esophagectomy with gastric pull-up and cervical esophagogastrostomy (logistic regression analysis; bootstrapping procedure)

Anastomotic dehiscence	Odds ratio	Std. Err	*t*	*p*	[95% Conf. Interval]	
Age	0.957	0.014	− 3.01	**0.003**	0.930	0.985
BMI	1.063	0.033	2.01	0.045	1.001	1.129
CRP 3rd postoperative day	1.004	0.002	2.21	**0.027**	1.000	1.008
Manual suturing	1.877	0.648	1.82	0.068	0.954	3.695
ASA	1.890	0.492	2,44	**0.015**	1.134	3.149
Retrosternal conduit transposition	1.644	0.597	1.37	0.171	0.807	3.351

*ASA* American Society of Anaesthesiologists performance score, *BMI* Body Mass Index, *CRP* C-reactive protein

Bold values are statistically significant for *P* values

**Fig. 1:**
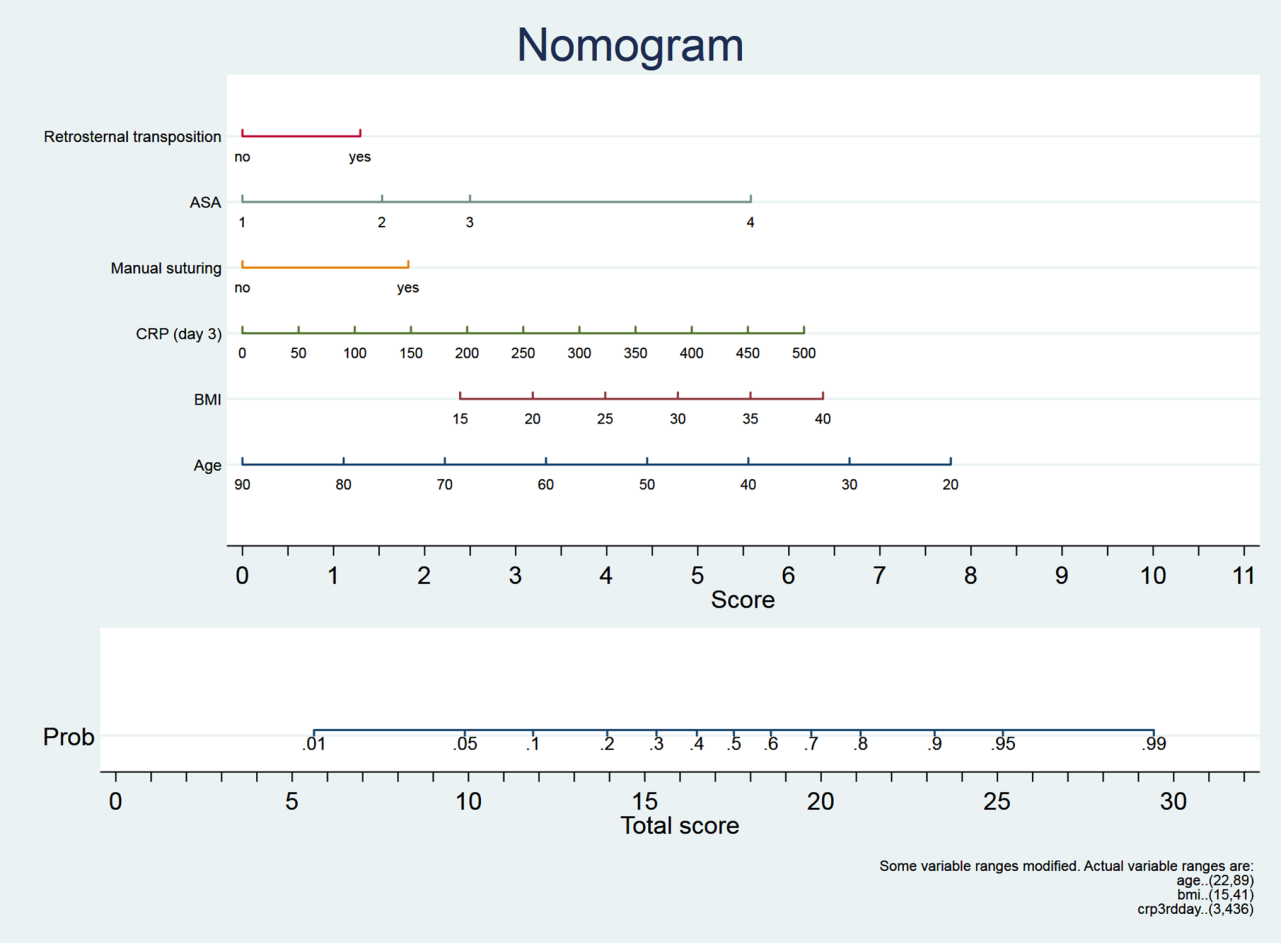
Nomogram illustrating the probability of development of an anastomotic leakage following cervical esophagogastrostomy. *ASA* American Society of Anaesthesiologists performance score, *CRP* C-reactive protein on the 3rd postoperative day, *BMI* Body Mass Index

Based on this multivariable logistic regression analysis, the nomogram for the determination of the individual patient’s risk was established. To use the nomogram, it is required to draw a vertical line from each of this six independent parameter mentioned above to the line named “Score”. After addition of those corresponding single score values, the total score is obtained. After marking this number on the line named “Total score”, the corresponding probability of development of an anastomotic dehiscence named “Prob” can be read above the Total Score Line (Fig. 1). For example, if a 60-year-old patient with BMI 35 and ASA 4 has to undergo retrosternal gastric pull-up with hand-sewn anastomosis, the total points would be 17.5, and the corresponding risk for postoperative anastomotic leakage would be 50%. ROC analysis revealed an Area under the ROC Curve = 0.7312.

## Discussion

This clinical analysis demonstrates that young patient’s age, retrosternal transposition of the gastric conduit, manual anastomotic suturing, elevated BMI, enhanced ASA score and high CRP levels on the 3rd postoperative day may serve as predictors for anastomotic leakage following cervical esophagogastrostomy. After combining these six parameters into a nomogram, the patient’s individual probability of development of an anastomotic dehiscence can be quantified.

In our series, cervical esophagogastrostomy was used routinely for esophageal reconstruction with the tubulated stomach. There is still discussion whether to apply cervical or intrathoracic anastomosis [21]. Several studies suggested increased leak rates following cervical anastomosis [2, 4, 12, 26], whereas two studies could not show a statistically significant difference [5, 27]. However, many surgeons still prefer the cervical esophagogastrostomy to the intrathoracic anastomosis. As mentioned above, this so-called Ivor-Lewis procedure has not been used during the present study due to two reasons. First, the oncological margin corresponding to the distance between the site of esophageal cancer and the cutting of the cervical esophagus is much wider in the case of cervical anastomosis. Second, the cervical anastomosis represents a safer and more beneficial procedure with low morbidity and mortality in particular in the case of anastomotic dehiscence as compared to intrathoracic anastomotic leakage with subsequent pleural empyema and septic reaction [4, 22, 23].

Nevertheless, impaired healing of the cervical anastomosis still impacts the quality of life, prolongs the hospital stay and may cause strictures with the need for repetitive dilation in up to 50% [6, 9]. In this context, the accurate prediction of those patients at high risk of anastomotic leakage, prior to the onset of clinical symptoms, would be beneficial in order to start tailored investigation and early intervention to prevent leakage development.

The overall rate of leakage in our collective was 18.4%. In our opinion, the high rate was mainly caused by the routine use of manual suturing (57%) during the first seven years of the observation period.

When analysed in detail, manual sutures had 24% leakage, whereas the semi-mechanical and circular stapled anastomoses had leakage not higher than 11%. Our results corroborate the findings reported by Kondra [28], similar results could be obtained by Okuyama [27].

The main reason for this relatively high leak rates in hand-sewn cervical esophagogastrostomy can be found in the impaired blood circulation in the mobilized and tubulated stomach leading to decreased tissue oxygen levels in the tip of the gastric conduit [9, 14]. Hand-sewn anastomosis may cause variations in needle spacing and unequal ligation strength resulting in impaired peri-anastomotic blood circulation. For this reason, this type of anastomosis represents a considerable risk factor for anastomotic leakage even in our collective of patients. In this context, several surgeons favour the partially stapled cervical esophagogastrostomy according to Orringer [9], which could lower the leak rate could significantly [1, 9, 28]. For the sake of completeness, it has to be mentioned that during the observation period of the present study the anastomotic type has changed according to the technical progress. However, the individual use of the anastomotic technique was done according to the underlying situation and was left at the discretion and the experience of the treating surgeon.

The significantly higher rate of leakage in obese patients may be caused by the abundance of mesenteric adipose tissue resulting in a bulky conduit that fits poorly into the mediastinum. As suggested by Briel, this may result in localized vascular insufficiency [13]. In this context, perfusion of the apex of the gastric conduit at the anastomotic site can also be jeopardized by undue tension on the suture line resulting in local hypoxia and impaired anastomotic healing [14]. Body habitus in heavier patients may contribute to technical difficulties with the cervical anastomosis resulting in subsequent anastomotic leakage [13]. However, we were able to support this assumption as we could identify an elevated BMI as a predictor of cervical anastomotic leakage.

Choice of retrosternal route of reconstruction has also been described as a risk factor for anastomotic dehiscence. Again, a reduction in blood flow is the underlying cause [9]. For anatomical reasons, the retrosternal route for gastric pull-up is about 2–5 cm longer than the orthotopic one. In addition, the sternoclavicular joint may impinge on a retrosternal conduit. It is possible that gastric pull-up via the retrosternal route may cause more mechanical stress to the stomach than the orthotopic route, resulting in impaired perfusion and reduced oxygen supply. In the course of a former study, we could demonstrate that the oxygen supply at the cervical esophagogastrostomy reached significantly higher levels after orthotopic than retrosternal gastric transposition (68.2 versus 24.6 mmHg; *p* < 0.001), [14].

This finding corroborates the results of our present study revealing the retrosternal transposition of the tubulated stomach as independent significant predictor of cervical anastomotic dehiscence.

Retrosternal transposition was particularly common in earlier years and less common in later years. Initially, it was preferred because our radiotherapists previously suggested that postoperative radiation would be better applicable when there is no gastric conduit in the original bed of the esophagus. So, for example, retrosternal transposition was used in 58% of patients in 2009, but only in 11% in 2017.

The fact that retrosternal transposition was significant in univariable but not in multivariable analysis may be due to the finding that there was a strong association of retrosternal transposition on the one hand and manual suturing on the other (manual suturing accounting for 86.7% in retrosternal and only for 42.6% in orthotopic replacement; χ^2^ = 67.8842, *p* < 0.001), and so manual suturing retained significance in the multivariable approach.

Elevated postoperative CRP levels have been described as independent predictors of leakage in esophagogastric anastomosis [15, 16]. We could confirm this assumption in the present study.

Only elevated levels of CRP on the 3rd postoperative day were significantly connected with the development of anastomotic dehiscence as it was demonstrated in multivariable testing. Elevated CRP levels on the third operative day may indicate ongoing inflammation, though we otherwise were unable to find a respective predictive value of other inflammatory markers, namely, lactate, white blood cell count, as stated by other investigators [15, 17]. However, we cannot rule out the possibility that CRP levels obtained on later days might have an even higher impact.

The ASA score depicts the estimated anaesthesiological risk of an intervention and mirrors the degree of patient’s comorbidity. In our collective of patients, high ASA scores corresponding to enhanced comorbidity were significantly connected with a higher rate of anastomotic leakage as shown by logistic regression analysis and multivariable analysis (Table 3 and Table 5). The findings of a large retrospective study with 654 patients confirm our results [2]. Recent data have shown that ischemia of the gastric conduit, which leads to anastomotic leakage, is more likely to occur in the presence of comorbid conditions.

These conditions, mostly impairment of the kidney function, cardiovascular disorders, diabetes, hypertension and chronic obstructive pulmonary disease, are known to compromise tissue perfusion and oxygenation with subsequently impaired anastomotic healing [13]. In this context, higher number of comorbidities have shown to serve as an independent risk factor for anastomotic dehiscence [1, 12, 13]. As far as smoking is concerned, we had to rely on data provided by the patients themselves, which might have been biased, which in turn would explain the lack of statistical relationship to anastomotic leak. Moreover, some authors identified the presence of induction therapy (preoperative chemotherapy and combined chemo-radiotherapy) as an independent risk factor for anastomotic leakage [2, 13, 17]. However, we could not corroborate this assumption in the present study.

Surprisingly, in the present study, the rate of anastomotic dehiscence was significantly higher in younger individuals. This finding is amazing since wound healing is expected to proceed better in younger than in older patients. Yet, one could hypothesize that younger patients tend to resume vigorous movements during postoperative mobilization earlier than older ones.

In this context, Pirmoazen and team had shown that an intentional flexion of the neck in the immediate postoperative period relieves tension on the anastomosis resulting in a considerable reduction of the rate of anastomotic leakage following cervical esophagogastrostomy [19]. However, this assumption has to be interpreted with caution due to the very small group of investigated patients.

Many efforts have been undertaken to develop new techniques that would prevent esophagogastric anastomotic leak [13, 29–31]. One feasible attempt might be the intentional delay of the postoperative oral intake in order to preserve the newly created anastomosis. Bolton and team have shown that planned delay of oral intake up to 12 days after surgery significantly reduces the cervical leakage rate from 23 to 3% [10]. Speicher and colleagues could significantly lower the leak rate from 14.5 to 4.2% when oral feeding was not started before the 15th postoperative day [11]. Though a long delay until the uptake of oral nutrition may indeed prevent anastomotic leakage in the majority of patients, it would entail a prolonged hospital stay. In this context, the decision for the delay to resume oral intake could be made based on the calculated risk factor of this nomogram as proposed by the present study.

By using this nomogram as a supportive measure in the postoperative course, the patient´s individual probability could be quantified which may help to take preventive measures before the onset of clinical symptoms. This would help us to treat patients for suspected anastomotic leak as early as possible and to arrange the appropriate follow-up treatment. These pre-operatively identified patients at high risk for anastomotic leakage should undergo focused clinical assessment with closer monitoring as usually done. For this reason, daily appraisal of the cervicotomy and daily check of the inflammatory laboratory parameters (leucocytes and CRP) should be done followed by advanced flexible endoscopy. In the case of incipient anastomotic leakage, immediate endoscopic and/or surgical treatment may be initiated.

However, the difference of the present study is that the nomogram is easier and more intuitive than existing methods. The nomogram includes both pre- and intraoperative parameters which have shown to contribute to the development of an anastomotic leakage. This current scoring method is able to clarify the combination of surgical factors and patient factors by focusing on those six simple but substantial parameters namely patient’s age, ASA, BMI, conduit transposition, anastomotic type and postoperative CRP.

The main advantages of this novel scoring tool are that this nomogram can be supplied very easily with these simply available parameters mentioned above. Furthermore, in the case of scheduled esophagectomy, very early identification of these patients at high risk for anastomotic leakage would be possible, even pre-operatively.

In those cases with high scoring, the treating surgeon may be alert, and closer monitoring of the affected patients should be initiated in the early postoperative period. The aims should be to detect the development of an anastomotic leakage as early as possible and to start appropriate preventive measures. However, these different endoscopic and surgical treatment options will be nearly the same usually applied in the case of manifest leakage but the time of starting these interventions makes the significant difference. In this context, early and timely initiation of postoperative preventive treatment is of utmost importance to inhibit the progress of anastomotic dehiscence and to accelerate anastomotic healing.

Finally, there are some limitations in this study which have to be mentioned. The study was retrospective, observational and conducted at a high-volume single institution. Therefore, a selection bias cannot be fully excluded within this heterogeneous cohort of patients. Moreover, we cannot rule out the presence of some residual confounding by factors that were not included in the analysis due to not being collected during data ascertainment. Furthermore, due to the limited number of cases, a split into a training set and a test set was not performed, which, however, in part was overcome by applying a jackknife procedure. Thus, larger prospective multicentric studies will have to be carried out in order to approve these preliminary results.

## Conclusion

Based on the findings of the present study, we can conclude that using the nomogram as a supportive measure in the perioperative management may help to identify these patients at high risk for development of an anastomotic leakage following cervical esophagogastrostomy. By initiation of an early diagnostic assessment and subsequently taking appropriate preventive measures, the development of a cervical anastomotic dehiscence might be precluded before the onset of clinical symptoms.
